# WISP-1 Promotes Epithelial-Mesenchymal Transition in Oral Squamous Cell Carcinoma Cells via the miR-153-3p/Snail Axis

**DOI:** 10.3390/cancers11121903

**Published:** 2019-11-29

**Authors:** An-Chen Chang, Ming-Yu Lien, Ming-Hsui Tsai, Chun-Hung Hua, Chih-Hsin Tang

**Affiliations:** 1 School and Medicine, China Medical University, Taichung 404, Taiwan; annone3212@gmail.com (A.-C.C.); minghsui@mail.cmuh.org.tw (M.-H.T.); 2 Division of Hematology and Oncology, Department of Internal Medicine, China Medical University Hospital, Taichung 404, Taiwan; d12604@mail.cmuh.org.tw; 3 Graduate Institute of Basic Medical Science, China Medical University, Taichung 404, Taiwan; 4 Department of Otolaryngology, China Medical University Hospital, Taichung 404, Taiwan; D3797@mail.cmuh.org.tw; 5 Graduate Institute of Biomedical Sciences, China Medical University, Taichung 404, Taiwan; 6 Graduate Institute of Clinical Medical Science, China Medical University, Taichung 404, Taiwan; 7 Chinese Medicine Research Center, China Medical University, Taichung 404, Taiwan; 8 Department of Biotechnology, College of Health Science, Asia University, Taichung 413, Taiwan

**Keywords:** OSCC, WISP-1, EMT, Snail, miR-153-3p

## Abstract

Around half of all patients with oral squamous cell carcinoma (OSCC) present with lymphatic metastasis, a strong predictor of poor survival. Improving survival rates depends on preventing the first step in the “invasion-metastasis cascade,” epithelial-to-mesenchymal transition (EMT), and developing antilymphangiogenesis therapies that antagonize lymphatic metastasis. The extracellular matrix-related protein WISP-1 (WNT1-inducible signaling pathway protein-1) stimulates bone remodeling and tumor progression. We have previously reported that WISP-1 promotes OSCC cell migration and lymphangiogenesis induced by vascular endothelial growth factor C (VEGF-C). This investigation sought to determine the role of WISP-1 in regulating EMT in OSCC. Our analysis of oral cancer data from The Cancer Genome Atlas (TCGA) database revealed significant and positive associations between levels of WISP-1 expression and clinical disease stage, as well as regional lymph node metastasis. We also found higher levels of WISP-1 expression in serum samples obtained from patients with OSCC compared with samples from healthy controls. In a series of in vitro investigations, WISP-1 activated EMT signaling via the FAK/ILK/Akt and Snail signaling transduction pathways and downregulated miR-153-3p expression in OSCC cells. Our findings detail how WISP-1 promotes EMT via the miR-153-3p/Snail axis in OSCC cells.

## 1. Introduction

The vast majority (an estimated 95%) of oral neoplasms are diagnosed as oral squamous cell carcinoma (OSCC) [1], which primarily affects the tongue (34.1%), palate (13.5%), buccal mucosa (13.3%), and floor of the mouth (12.2%) [2,3]. Generally, the clinical features of OSCC show ulcerated lesions with central necrotic areas and rolled up margins [4]. Surgery, chemotherapy, and radiotherapy are standard treatment options in early-stage OSCC, although over half of all such treated patients die within 5 years of treatment [5], while 5-year survival is only around 12% for patients who present with late-stage disease; most die within the first 30 months after diagnosis [6]. Poor prognosis is also associated with local recurrence or the development of distant metastases following completion of treatment [5]. It is therefore crucial that we understand the underlying mechanisms of metastasis in OSCC to improve its treatment and survival rates.

The epithelial-to-mesenchymal transition (EMT) phenomenon describes a process whereby the migratory capacity and invasiveness of epithelial cells is enhanced via the loss of cell–cell adhesions and polarity, enabling them to transition into cells with a mesenchymal phenotype [7,8]. During this highly dynamic process, EMT activation decreases levels of epithelial markers (e.g., E-cadherin) and increases levels of mesenchymal markers (e.g., N-cadherin, vimentin, and fibronectin) in cancers [9,10]. Tumor hypoxia and tumor-associated proteins, such as hepatocyte growth factor (HGF), transforming growth factor β (TGF-β), platelet-derived growth factor (PDGF), fibroblast growth factor (FGF), and epidermal growth factor (EGF), appear to be responsible for the induction or functional activation of EMT in cancer cells [8,11,12,13,14]. Importantly, EMT is considered to be the initial step in the “invasion-metastasis cascade” [7,8]. EMT is largely regulated by a core set of EMT-activating transcription factors (EMT-TFs) including Slug, Twist, Snail, and ZEB1/2, amongst others [15]. Snail, one of the most closely studied EMT-TFs, binds to the promoter region of E-cadherin E-boxes to repress the transcription of E-cadherin and thus downregulates cell–cell adhesion and promotes mesenchymal morphogenesis [16,17]. EMT-activated cancer cells become highly mobile, more stem cell-like (self-renew and differentiate into different tumor cell types), and are better able to infiltrate immune cell populations and become treatment-resistant [18,19,20].

MicroRNAs (miRNAs) are closely associated with EMT progression in the cellular signaling pathway [21,22]. By binding to their complementary sequences in the 3’-untranslated regions (3’-UTRs) at the miRNA recognition elements (MREs) of target mRNAs, miRNAs effectively degrade mRNAs and/or inhibit their translation and subsequently regulate EMT functioning, invasiveness, and metastasis of epithelial cancer cells [23]. Several miRNAs (including miR-143-3p, miR-300, miR-99a-5p, miR-99b-3p, let-7c-5p, miR-100-5p, and miR-125b-5p) have been implicated in OSCC proliferation, migration, and invasion [24,25] and, in particular, low levels of miR-143-3p, miR-300 and miR-99a-5p expression are significantly associated with poor overall survival (OS) in patients with OSCC [24,25,26]. Moreover, miR-300 inhibits EMT by significantly increasing mRNA levels of E-cadherin and significantly decreasing mRNA levels of vimentin, N-cadherin, matrix metalloproteinase-2 (MMP-2), and Snail1 in OSCC cells [25]. The use of OSCC-associated miRNA has been considered as a potential tool for predicting the presence of OSCC from oral swirls or plasma [27,28]. However, their mechanisms of EMT in OSCC remain unclear. More research is needed to evaluate the role of miRNA-regulated EMT in OSCC.

Tumor-derived growth factors are critical for advancing tumor progression and enabling tumors to evade immune surveillance, and for driving the EMT process and metastasis to distant organs [29,30]. We have previously demonstrated that OSCC-secreted WNT1-inducible signaling pathway protein 1 (WISP-1) promotes epithelial cell migration and VEGF-C-dependent lymphangiogenesis [31,32]. WISP-1 stimulates VEGF-C expression and lymphangiogenesis by suppressing miR-300 expression via the integrin αvβ3/integrin-linked kinase (ILK)/Akt signaling pathway [31]. WISP-1, also known as CCN4, contributes to tumorigenesis [33], is a predictor of poor survival [34] and is expressed at high levels not only in OSCC, but also in several other types of cancer, including esophageal cancer, pancreatic ductal adenocarcinoma, and osteosarcoma [34,35,36]. However, it is unclear as to whether WISP-1 positively influences the EMT process in OSCC. Our study shows that WISP-1 mRNA expression is significantly associated with clinical disease stage and regional lymph node metastasis. According to in vitro evidence, WISP-1 promotes Snail activation via the focal adhesion kinase (FAK)/ILK/Akt signaling transduction pathway, leading to EMT activation. Another important function of WISP-1 is its inhibition of miR-153-3p mRNA expression, preventing miR-153-3p from targeting the 3’-UTR of Snail, while miR-153-3p mimic prevents this process. Our results provide insights into how WISP-1 mediates EMT via the miR-153-3p/Snail axis in OSCC.

## 2. Materials and Methods

### 2.1. Cell Culture

The human OSCC cell line SCC4 was purchased from the Bioresource Collection and Research Center (BCRC; Hsinchu, Taiwan). Cells were grown in a 1:1 mixture of GIBCO^®^ Dulbecco’s Modified Eagle Medium: Nutrient Mixture F-12 (DMEM/F-12) media containing 10% fetal bovine serum (FBS; Gibco, Thermo Fisher Scientific; Waltham, MA, USA). SCC4 cells were subcultured at ~80% of cell confluence.

### 2.2. Preparation of Serum Specimens

This project was approved by the Institutional Review Board of China Medical University Hospital (CMUH105-REC3-042). From 2014 through 2016, all enrolled study participants provided written informed consent before their blood was drawn in China Medical University Hospital. Serum specimens were isolated from blood samples by centrifuging and were stored at −80 °C until use.

### 2.3. Wound Healing

SCC4 cells were seeded onto 6-well plates and allowed to reach ~90% confluence, before a monolayer of SCC4 cells was manually scratched with a pipette tip to create extended wound-like scratches in each well. Detached cells were removed by phosphate-buffered saline (PBS) wash. SCC4 cells were then treated with different concentrations of human recombinant WISP-1 protein (PeproTech; Rocky Hill, NJ, USA). After 24 h of migration, microscope images of living cells were acquired (Zeiss; Oberkochen, Germany) and the width of the gap in each scratch was measured by ImageJ software.

### 2.4. Western Blot 

Preparation of cell lysates from SCC4 cells in 6-well plates and protein concentration was determined for each cell lysate using the BCA Protein Assay Kit (Thermo Fisher Scientific; Waltham, MA, USA). An amount of 30–50 μg of protein was loaded into each well containing 10% SDS-PAGE gel then transferred to polyvinylidene difluoride (PVDF) membranes (EMD Millipore; Temecula, CA, USA) for Western blot analysis, according to our previous research [32,37]. Membranes were probed with their respective antibodies; anti-ILK (Santa Cruz; Dallas, TX, USA), anti-GSK3β(Santa Cruz; Dallas, TX, USA) anti-FAK (Cell Signaling; Danvers, MA, USA), anti-Akt (Cell Signaling; Danvers, MA, USA), anti-Snail (Santa Cruz; Dallas, TX, USA), anti-Twist (Santa Cruz; Dallas, TX, USA), anti-E-cadherin (Abcam; Cambridge, MA, USA), and anti-α-tubulin (Santa Cruz; Dallas, TX, USA). Immunoblot images were acquired using the ImageQuant™ LAS 4000 biomolecular imager (GE Healthcare Life Sciences; Pittsburgh, PA, USA).

### 2.5. Quantitative Real-Time Polymerase Chain Reaction (qPCR)

Total RNA was extracted from SCC4 cells in the 6-well plates and a GE NanoVue Plus spectrophotometer (GE Healthcare Life Sciences; Pittsburgh, PA, USA) determined RNA concentrations for each sample. The M-MLV RT kit (Invitrogen, Thermo Fisher Scientific; Waltham, MA, USA) and the Mir-X™ miRNA First-Strand Synthesis kit (Clontech; Mountain View, CA, USA) were used to perform reverse transcription of total RNA into complementary DNA (cDNA). PCR amplification was performed as previously described [32,38,39].

### 2.6. Immunoprecipitation (IP)

Supernatants were prepared from total cell lysates (non-denaturing) and co-precipitated with anti-ILK antibody (10 μL; Santa Cruz; Dallas, TX, USA) and Protein G beads (30 μL; EMD Millipore; Temecula, CA, USA) for 12 h at 4 °C under gentle rotation. After immunoprecipitation, samples were washed 3 times with iced lysis buffer and proteins in each sample were denatured for 5 min at 95 °C. Samples were stored at −20 °C until they underwent Western blot analysis for specific protein expression.

### 2.7. ELISA Assay

Sixty-two serum samples in total were obtained from patients with OSCC and 11 serum samples from healthy controls. Serum WISP-1 was assayed using the WISP-1 ELISA kit (ASIA BIOSCIENCE; Taipei, Taiwan), according to the manufacturer’s procedure.

### 2.8. Immunofluorescence (IF)

SCC4 cells were prepared on 12-mm coverslips in 24-well plates, then treated with WISP-1, using previously established procedures [32]. Levels of fluorescence expression were detected using fluorescence microscopy (Zeiss; Oberkochen, Germany).

### 2.9. Plasmid Construction and Luciferase Assays

Wild-type and mutant plasmids of human Snail 3’-UTR containing the miR-153-3p binding site were constructed by MDBio, Inc. (Taipei,Taiwan). SCC4 cells were transfected with plasmid using Lipofectamine 2000 (Invitrogen; Thermo Fisher Scientific; Waltham, MA, USA) for 24 h, then treated for an additional 24 h with WISP-1. Luminescence expression was analyzed using the Dual-Luciferase Reporter Assay System (Promega Corporation; Madison, WI, USA), as previously described [40].

### 2.10. Meta-Analysis of Microarray Datasets From The Cancer Genome Atlas (TCGA) Database

Using the TCGA dataset of head and neck cancers, we identified 310 patients with oral tumors diagnosed in the tongue, alveolar ridge, oral cavity, hard palate, floor of the mouth, or buccal mucosa. Levels of WISP-1, E-cadherin and Snail mRNA expression were analyzed for each tumor sample.

### 2.11. Statistics

All quantified results were calculated using GraphPad Prism 5.0 (GraphPad Software. Inc; San Diego, CA, USA) or SigmaPlot 10.0 software (Systat Software, Inc; San Jose, CA, USA) and are presented as the mean ± standard error of the mean (SEM). The Student’s *t*-test was used to compare the means of two experimental groups. We performed a one-way ANOVA followed by Bonferroni’s post hoc comparison tests on results from statistical comparisons involving more than 2 groups. In all cases, *p* < 0.05 was considered statistically significant.

## 3. Results

### 3.1. Clinicopathologic Characteristics of WISP-1 in Oral Cancer Based on the TCGA Database

**Figure 1:**
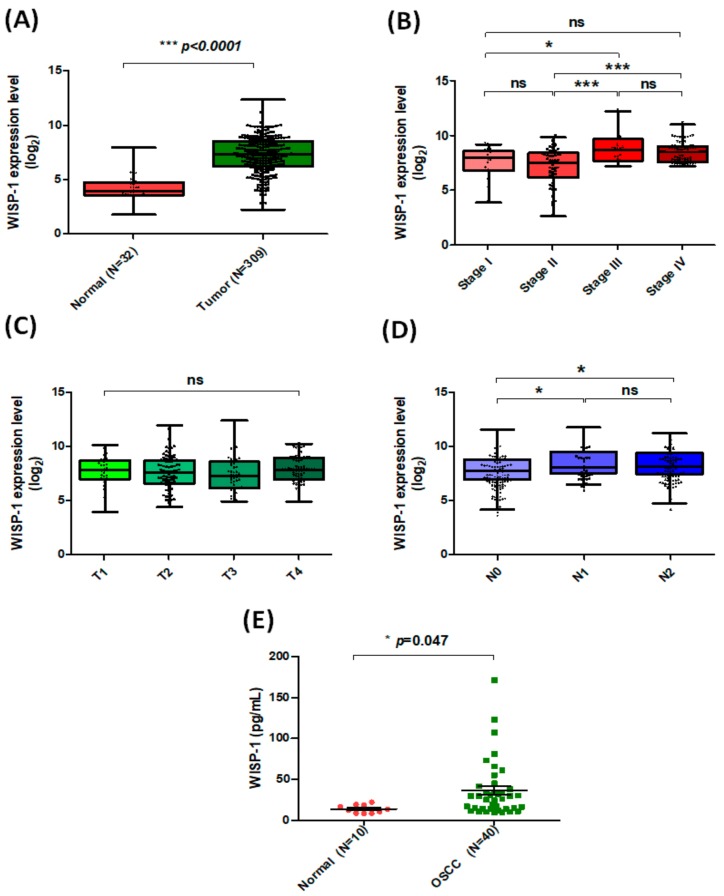
Levels of WISP-l expression correlate with clinicopathologic features of oral cancer. (**A**) WISP-1 mRNA expression in tumor tissue and adjacent normal tissue was analyzed using records from The Cancer Genome Atlas (TCGA) database. Median levels (ranges) of WISP-1 expression in normal and tumor tissue samples: 4.168 (1.794–7.998) and 7.286 (2.257–12.416), respectively; log_2_(fold-change): 3.118. (**B**–**D**) Analyses of the TCGA records revealed the following median levels (ranges) of WISP-1 expression according to disease classification: stage I, 7.688 (4.013–9.373); stage II, 7.225 (2.756–10.037); stage III, 9.036 (7.367–12.416); stage IV, 8.622 (7.352–11.203); log_2_(fold-change): stage I vs. stage III: 1.348; stage II vs. stage III: 1.811; stage II vs. stage IV: 1.397; according to tumor status (median levels (range) of WISP-1 in T1–T4: T1: 7.778 (4.013–10.174); T2: 7.631 (4.464–11.978); T3: 7.499 (4.995–12.416); T4: 7.957 (4.973–10.275)) and according to regional lymph nodes (median levels (ranges) of WISP-1 in N0-N2: N0: 7.267 (3.621–11.007); N1: 7.985 (5.927–11.203); N2: 7.842 (4.196–10.674); log_2_(fold-change): N0 vs. N1: 0.718, N0 vs. N2: 0.575). (**E**) The ELISA assay was used to measure WISP-1 levels in serum specimens. Results are expressed as the mean ± SEM. * *p* < 0.05, ** *p* < 0.01, *** *p* < 0.005, NS *p* *>* 0.05 compared with the control group.

We have previously demonstrated that OSCC-derived WISP-1 increases its motility and lymphangiogenesis to facilitate lymph node metastasis [31,32]. In this study, we used samples from the TCGA database to explore WISP-1 mRNA tissue expression and its clinical significance. Levels of WISP-1 mRNA expression were much higher in tumor tissue than in adjacent normal tissue (Figure 1A) and significant associations were observed between high levels of WISP-1 expression and clinical disease stage (Figure 1B) and regional lymph node metastasis (Figure 1D), but there was no such effect on clinical tumor status (Figure 1C). We also found higher levels of WISP-1 expression in serum samples obtained from patients with OSCC compared with healthy controls (Figure 1E). These findings indicate that WISP-1 appears to be overexpressed in OSCC and associated with lymph node metastasis. Similarly to our previous observations, WISP-1 promotes VEGF-C-dependent lymphangiogenesis to advance lymph node metastasis [31].

### 3.2. WISP-1 Downregulates E-Cadherin Expression to Advance Mesenchymal Morphogenesis in OSCC Cells

**Figure 2:**
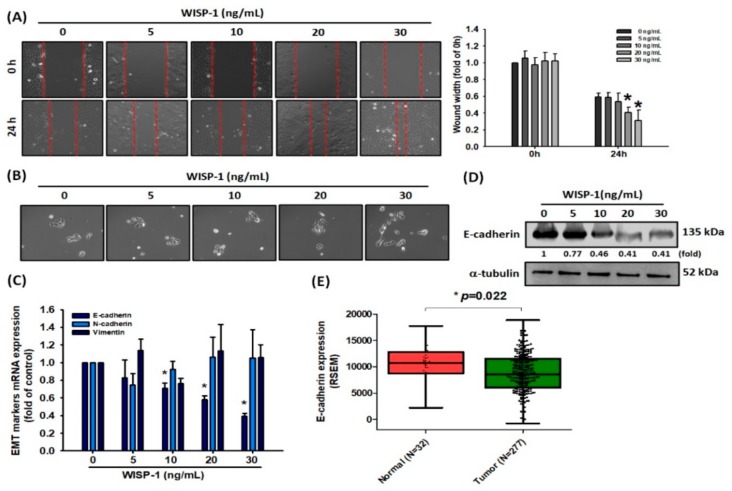
WISP-1 induces epithelial-to-mesenchymal transition (EMT) functioning via E-cadherin downregulation. (**A**,**B**) SCC4 cells were treated with different concentrations of WISP-1 (0–30 ng/mL) for 24 h; cell migration and cell-scattering phenotype were analyzed by wound healing and scatter assays, respectively. Images of living cells were captured by microscope and the width of the gap in each scratch was measured by ImageJ software. (**C**,**D**) Cells were incubated with different concentrations of WISP-1 (0–30 ng/mL) for 24 h and EMT marker expression was evaluated by qPCR and Western blot assay. The α-tubulin content was used to normalize for levels of E-cadherin protein. (**E**) E-cadherin mRNA expression in tumor tissue and adjacent normal tissue was determined in TCGA database records. Mean levels (ranges) of mRNA expression in normal and tumor samples, respectively: 10,660 (2858.6–17,763.1), 9095 (4.929–18,812.1); log_2_(fold-change): –0.229. Results are expressed as the mean ± SEM. * *p* < 0.05 compared with the control group.

Cancer cells gain migratory and invasive properties through active EMT functioning, which occurs during wound healing and in the initiation of cancer metastasis [7,8]. We therefore hypothesized that OSCC-derived WISP-1 potentially affects cancer cell migration through the EMT process. Firstly, we measured WISP-1 basal expression in different OSCC cell lines. In our analysis of the Cancer Cell Line Encyclopedia (CCLE) database, levels of SCC4 expression were higher than CAL27 and lower than those of SCC9 expression (Supplementary Figure S1A). Treatment of SCC4 cells with recombinant human WISP-1 protein revealed that WISP-1 induces cell motility in the wound healing assay (Figure 2A) and promotes the transitioning of SCC4 cells from the epithelial to the mesenchymal phenotype (Figure 2B). To explore the molecular mechanism of WISP-1 in EMT function, we treated SCC4 cells with WISP-1 for 24 h and then assessed the levels of EMT marker mRNA expression. We found that WISP-1 significantly inhibited epithelial marker E-cadherin mRNA and protein expression (Figure 2C,D), but did not affect levels of N-cadherin and vimentin expression (Figure 2C). Interestingly, further treatment of cells with WISP-1 for 48 or 72 h revealed a loss of inhibitory effects on E-cadherin expression (Supplementary Figure S1B). This suggests that WISP-1 has short-term effects on the inhibition of E-cadherin expression. A subsequent analysis of E-cadherin mRNA expression in TCGA tumor tissue identified significantly lower levels of E-cadherin expression in tumor tissue compared with adjacent normal healthy tissue (Figure 2E), although a subsequent analysis of TCGA data revealed a weak correlation between WISP-1 and E-cadherin (Supplementary Figure S2A). Thus, WISP-1 appears to promote cell motility and activate EMT functioning by inhibiting E-cadherin expression in OSCC cells.

### 3.3. The WISP-1/Integrin αvβ3 Axis Contributes to Regulating EMT Activation

**Figure 3:**
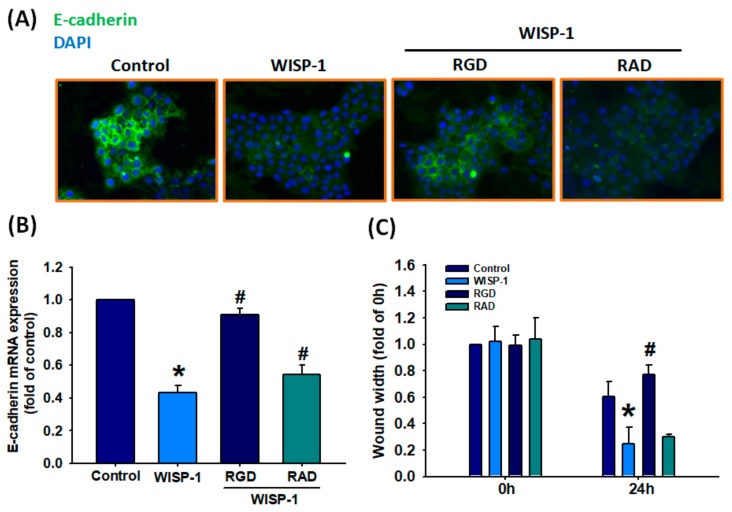
WISP-1 stimulates the EMT process via the integrin αvβ3 receptor in oral squamous cell carcinoma (OSCC) cells. (**A**,**B**) SCC4 cells were pretreated with Arg–Gly–Asp (RGD) (100 nM) or Arg–Ala–Asp (RAD) (100 nM) for 30 min, then stimulated with WISP-1 for 24 h. E-cadherin expression was examined by IF and qPCR assays. (**C**) Cells were pretreated with RGD or RAD for 30 min then stimulated with WISP-1 for 24 h, and cell migration was examined by the wound healing assay. Results are expressed as the mean ± SEM. * *p* < 0.05 compared with controls; # *p* < 0.05 compared with the WISP-1-treated group.

WISP-1 is characterized by four structurally distinct domains: the insulin-like growth factor-binding protein (IGFBP) domain, the von Willebrand factor type C repeat (vWC) domain, the thrombospondin type 1 repeat (TSP1) domain, and the cysteine-rich C-terminal module (CT) domain [41], among which the vWC, TSP1, and CT domains reportedly contain putative integrin recognition sites of WISP-1 [42]. According to previous research, WISP-1 directly binds to integrin αvβ3 to activate downstream signaling proteins and stimulate cell motility [32,43]. In this study, we found that the cyclic Arg–Gly–Asp (RGD) peptide, as a target epitope of integrin αvβ3, blocked WISP-1-mediated E-cadherin protein and mRNA expression. The cyclic Arg–Ala–Asp (RAD), a negative control peptide for cyclic RGD, had no effect on WISP-1-mediated E-cadherin expression (Figure 3A,B). We also found that RGD inhibited WISP-1-induced cell migration (Figure 3C). Thus, WISP-1 regulates EMT functioning via the integrin αvβ3 receptor in OSCC.

### 3.4. The Role of FAK and ILK Signaling Pathways in WISP-1-Activated EMT Functioning

**Figure 4:**
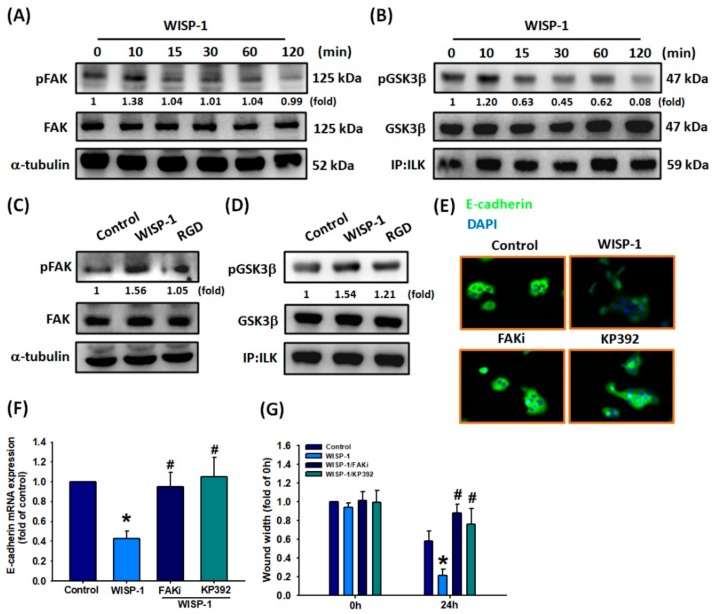
Involvement of FAK and ILK in WISP-1-regulated EMT functioning. (**A**,**B**) SCC4 cells were incubated with WISP-1 (30 ng/mL) for the indicated time intervals; FAK and ILK activation was examined by Western blot assay. (**C**,**D**) Cells were pretreated with RGD (100 nM) for 30 min then treated with WISP-1 for 10 min; FAK and ILK activation was examined by Western blot assay. FAK and GSK3β content was used to normalize for pFAK and pGSK3βlevels. (**E**,**F**) Cells were pretreated for 30 min with a FAKi (10 M) and KP392 (10 M), prior to incubation with WISP-1 for 24 h. E-cadherin expression was examined by IF and qPCR assays. (**G**) Cells were pretreated for 30 min with a FAKi (10 M) and KP392 (10 M), then incubated with WISP-1 for 24 h. Cell migration was examined by the wound healing assay. Results are expressed as the mean ± SEM. * *p* < 0.05 compared with controls; # *p* < 0.05 compared with the WISP-1-treated group.

Integrins mediate signaling by interacting with intracellular protein kinases, such as focal adhesion kinase (FAK) and integrin-linked kinase (ILK), transducing their intracellular signals through the cytoplasmic domain of the β integrin subunit and thereby regulating cell proliferation, survival, differentiation, and migration [44,45]. It is established that upon integrin activation, the receptor subsequently activates both FAK and ILK phosphorylation, which activate the downstream signaling pathway and phosphorylation levels are restored to baseline levels. Similarly, we detected high levels of FAK and ILK phosphorylation after OSCC cells were incubated for 10 min with WISP-1 30 ng/mL (Figure 4A,B). Subsequently, FAK and ILK phosphorylation activated the downstream signaling pathway. In a series of experiments, levels of FAK and ILK phosphorylation were subsequently stabilized, with restoration to baseline levels after a prolonged period (i.e., between 15–120 min). Pretreating the cells with RGD blocked WISP-1-induced FAK and ILK activation (Figure 4C,D). Pretreatment of OSCC cells with an FAK inhibitor (FAKi) or ILK inhibitor (KP392) abolished WISP-1-regulated suppression of E-cadherin protein and mRNA expression, as well as cell migration (Figure 4E–G). Thus, WISP-1 induces EMT functioning and OSCC cell migration via the integrin αvβ3/FAK/ILK signaling pathway.

### 3.5. Effect of Akt Activation in WISP-1-regulated EMT Functioning

**Figure 5:**
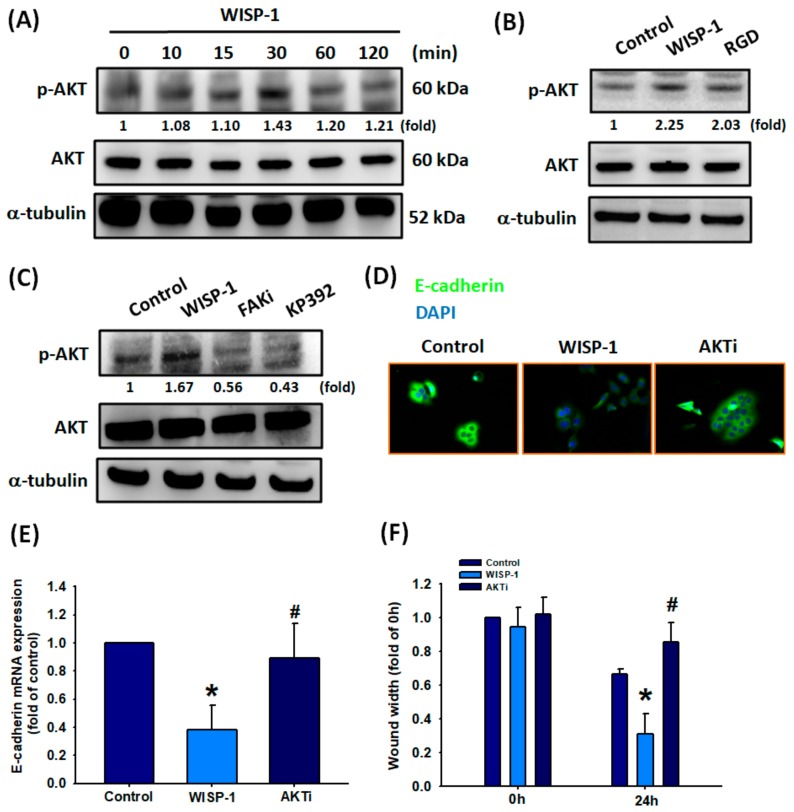
WISP-1 induces Akt phosphorylation via the integrin αvβ3/FAK/ILK signaling pathway. (**A**) SCC4 cells were incubated with WISP-1 (30 ng/mL) for the indicated time intervals; levels of Akt phosphorylation were examined by Western blot assay. (**B**,**C**) Cells were pretreated with RGD (100 nM), a FAKi (10 M), or KP392 (10 M) for 30 min, then incubated with WISP-1 for 30 min. Akt activation was examined by Western blot assay. Akt protein was used to normalize for pAkt levels. (**D**–**F**) Cells were pretreated for 30 min with Akti (10 M), prior to incubation with WISP-1 for 24 h. E-cadherin expression was examined by IF and qPCR assays. Cell migration was examined by the wound healing assay. Results are expressed as the mean ± SEM. * *p* < 0.05 compared with the control group; # *p* < 0.05 compared with the WISP-1-treated group.

Activation of FAK and ILK by integrins regulates and promotes the phosphorylation of downstream effectors, such as Akt (protein kinase B; PKB), which is responsible for cell survival and invasion [46]. In our study, we detected high levels of Akt activation after 30 min of WISP-1 treatment in OSCC cells (Figure 5A). According to our time course experiment, we observed high levels of pAkt expression over 10–120 min, with the highest value recorded at 30 min, in comparison with levels of pFAK and ILK expression (Figure 4A,B and Figure 5A). Our results provide good evidence showing that Akt is a downstream protein of FAK and ILK. Pretreatment of cells with RGD, FAKi, or KP392 interrupted WISP-1-promoted Akt phosphorylation (Figure 5B,C). We then used an Akt inhibitor (Akti) to observe the role of Akt in the regulation of WISP-1-mediated EMT functioning. We found that Akti prevented WISP-1-induced reductions in E-cadherin protein and mRNA expression, and WISP-1-induced migration of OSCC cells (Figure 5D–F). Thus, Akt protein is involved in WISP-1-mediated EMT functioning.

### 3.6. Upregulation of Snail in OSCC Cells Following WISP-1 Treatment

**Figure 6:**
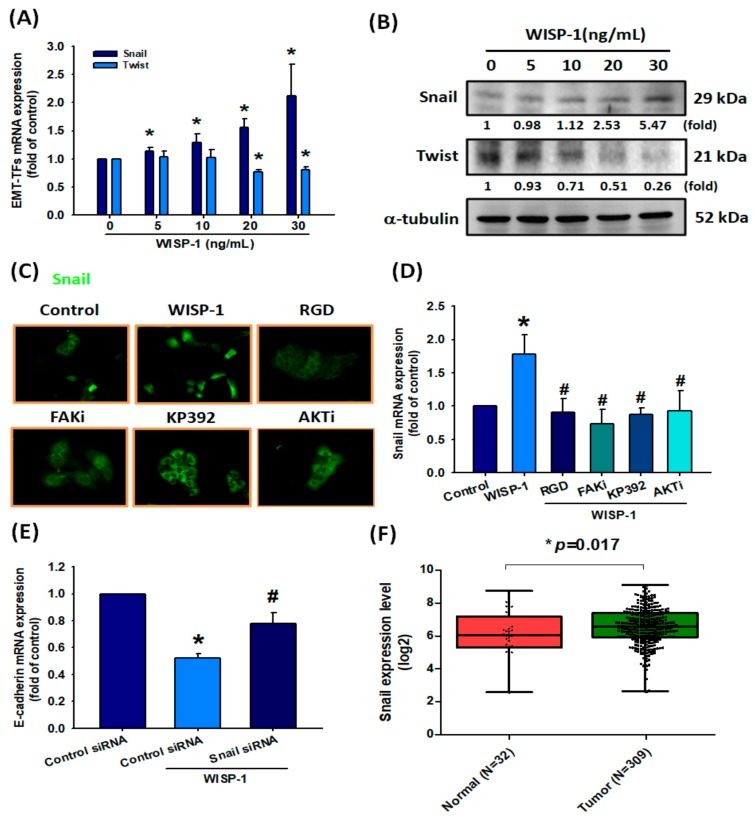
WISP-1 induces EMT function by Snail up-regulation in OSCC. (**A**,**B**) Cells were incubated with different concentrations of WISP-1 (0–30 ng/mL) for 24 h, then Snail and Twist expression were examined by qPCR and Western blot assays. The α-tubulin protein was used to normalize for levels of Snail and Twist. (**C**,**D**) Cells were pretreated for 30 min with RGD, FAKi, KP392, or Akti, prior to incubation with WISP-1 for 24 h. Snail translocation into the nucleus was examined by IF assay. Snail mRNA expression was examined by qPCR assay. (**E**) Cells were transfected with Snail siRNA (10 μM) or control siRNA (10 μM) for 24 h then stimulated with WISP-1 for 24 h; E-cadherin mRNA expression was examined using the qPCR assay. (**F**) Snail mRNA expression in tumor tissue and adjacent normal tissue was analyzed using records from the TCGA database. Mean levels (ranges) of Snail mRNA expression in normal and tumor samples, respectively: 6.064 (2.53–8.719), 6.555 (2.594–9.079); log_2_(fold-change): 0.491. Results are expressed as the mean ± SEM. * *p* < 0.05 compared with controls; # *p* < 0.05 compared with the WISP-1-treated group.

The transcriptional repression of E-cadherin can be orchestrated by EMT-TFs through binding to E-boxes present in the E-cadherin promoter [47]. We, therefore, assessed Snail and Twist expression in SCC4 cells following 24 h of WISP-1 treatment. We found that WISP-1 dose-dependently induced Snail protein and mRNA expression and slightly reduced levels of Twist expression (Figure 6A,B). An analysis of the role of Snail in the signaling pathway of WISP-1-inhibited E-cadherin expression revealed that WISP-1-induced Snail mRNA expression and nuclear translocation was inhibited by specific inhibitors of RGD, FAKi, KP392, or Akti (Figure 6C,D). To confirm that Snail is a critical inhibitor of E-cadherin via WISP-1 treatment, SCC4 cells were transfected with Snail siRNAs and E-cadherin mRNA expression was measured by qPCR assay. WISP-1-induced decreases in E-cadherin expression were abolished by Snail siRNAs (Figure 6E). Levels of Snail mRNA in oral cancer tissue were significantly upregulated compared with adjacent normal tissue samples (Figure 6F). Moreover, WISP-1 expression was positively correlated with Snail mRNA in human OSCC samples from the TCGA database (Supplementary Figure S2B). Thus, WISP-1 promotes EMT functioning in OSCC cells via the Snail EMT-TF.

### 3.7. Involvement of hsa-miR-153-3p in Regulating EMT Function

**Figure 7:**
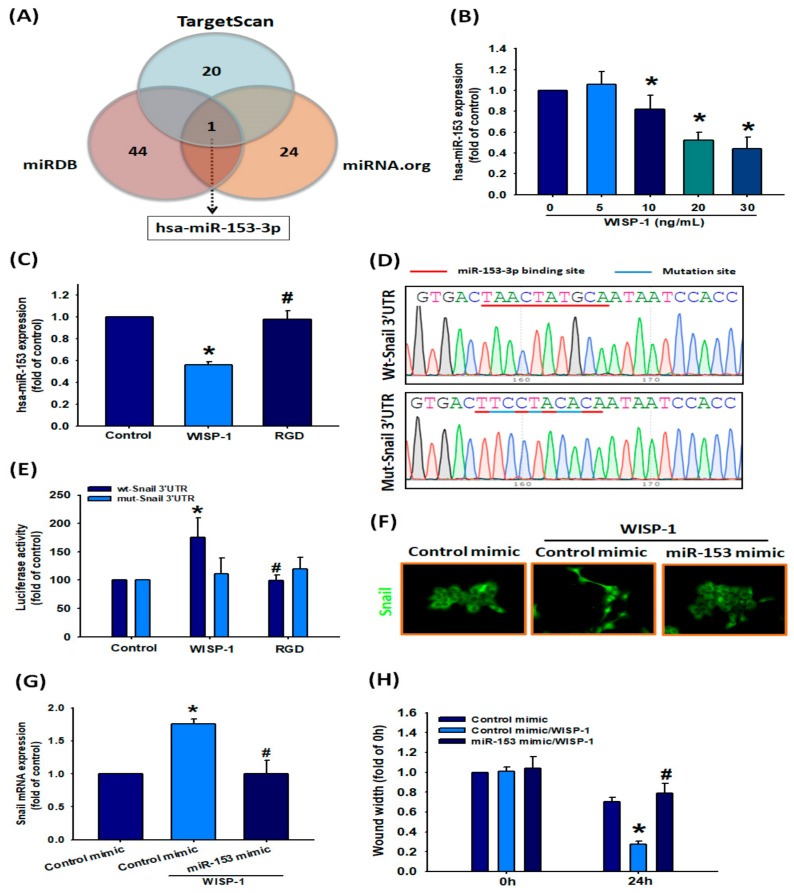
miR-153-3p inhibition is involved in WISP-1-induced EMT activation. (**A**) MiRNA target prediction program software was used to identify miRNAs that potentially bind to the Snail 3’-UTR. (**B**) After incubating cells with different concentrations of WISP-1 (0–30 ng/mL) for 24 h, miR-153-3p expression was examined by qPCR. (**C**) Cells were pretreated with RGD (100 nM) for 30 min then incubated with WISP-1 for 24 h; miR-153-3p expression was examined by qPCR. (**D**) The wild-type or mutant Snail 3’-UTRs containing the miR-153-3p binding site were inserted into the pmirGLO vector. (**E**) Cells were transfected with wt-Snail-3’-UTR (1 ug/uL) or mut-Snail-3’-UTR plasmid (1 ug/uL) for 24 h then stimulated with RGD for 30 min followed by WISP-1 treatment for 24 h; luciferase activities were measured. (**F**,**G**) Cells were transfected with miR-153-3p mimic (100 nM) or control mimic (100 nM) for 24 h then stimulated with WISP-1 for 24 h; Snail translocation into the nucleus was examined by IF assay. Snail mRNA expression was examined by qPCR assay. (**H**) Cells were transfected with miR-153-3p mimic (100 nM) or control mimic (100 nM) for 24 h; cell migration was examined using the wound healing assay. Results are expressed as the mean ± SEM. * *p* < 0.05 compared with controls; # *p* < 0.05 compared with the WISP-1-treated group.

Noncoding miRNAs regulate EMT functioning in clear cell renal cell carcinoma and lung cancer [21,22], although their mechanisms underlying WISP-1-mediated EMT functioning in OSCC have been uncertain up until now. This study identified that WISP-1 promotes EMT activation via Snail. Next, we sought to determine whether specific miRNAs are involved in WISP-1-induced inhibition of Snail expression. Analysis of three miRNA target prediction programs (i.e., TargetScan, miRDB, and miRNA.org) confirmed that miR-153-3p directly targets the 3’-UTR region of Snail (Figure 7A). WISP-1 treatment of SCC4 cells significantly downregulated levels of miR-153-3p expression via the integrin αvβ3 receptor (Figure 7B,C). To confirm that miR-153-3p directly binds to the 3’-UTR of Snail and inhibits Snail mRNA translation, we constructed the wild-type and mutant binding sites of Snail-3’-UTR luciferase plasmids (Figure 7D). We found that WISP-1 increased luciferase activity in the wt-Snail-3’-UTR plasmid via integrin αvβ3, but did not affect the mut-Snail-3’-UTR plasmid (Figure 7E). We next examined the role of miR-153-3p in the mechanism of WISP-1-activated EMT functioning in OSCC cells. Transfection of SCC4 cells with miR-153-3p mimic diminished WISP-1-promoted Snail nuclear translocation and mRNA expression, as well as cell migration (Figure 7F–H). According to our data, miR-153-3p inhibits Snail protein expression by integrating with the 3’-UTR region of Snail mRNA; however, WISP-1 inhibits this phenomenon.

## 4. Discussion

Standard treatments for OSCC include surgery of the primary lesion, radiotherapy, and chemotherapy, which are helpful in early-stage disease, although these approaches fail to benefit the majority of patients with the OSCC, who are diagnosed with late-stage disease (stages III or IV) and typically develop local recurrence or distant metastases [48,49]. It is, therefore, essential that we understand the underlying causes of tumor metastasis so that existing treatment for late-stage OSCC can be improved. In this study, we found significantly higher levels of WISP-1 expression in OSCC tissue compared with adjacent normal healthy tissue and we observed positive associations between WISP-1 expression, clinical disease stage, and lymph node metastasis. We also found that WISP-1 increases mesenchymal morphogenesis and cell motility by downregulating E-cadherin via the miR-153-3p/Snail axis in OSCC cells, suggesting that WISP-1 could serve as a novel therapeutic target in OSCC metastasis.

Previous research has highlighted the importance of the role played by WISP-1 and *WISP-1* polymorphisms in OSCC [50,51,52]. For instance, not only does WISP-1 increase tumor cell invasion and inhibit apoptosis in the human OSCC SCC-1483 cell line [50], but also, certain *WISP-1* single-nucleotide polymorphisms increase the susceptibility to OSCC and these associations are compounded by smoking and betel quid chewing [51]. Moreover, researchers have reported finding that *WISP-1* hypomethylation is associated with lymph node metastasis in patients with OSCC [52]. All this evidence is confirmed by our study findings showing significantly higher levels of WISP-1 expression in OSCC tissue compared with adjacent normal healthy tissue. We also observed positive associations between WISP-1 expression, clinical disease stage, and lymph node metastasis. Finally, we found that WISP-1 increases mesenchymal morphogenesis and cell motility by downregulating E-cadherin via the miR-153-3p/Snail axis in OSCC cells, suggesting that WISP-1 could serve as a novel therapeutic target in OSCC metastasis.

EMT was originally recognized as a feature of embryonic development [53]; EMT also plays a central role in cancer progression and metastasis when malignant cells invade host tissues and disseminate to distant organs [54]. Human OSCC develops when epithelial cells are affected by permanent high-risk behaviors, such as smoking and excessive drinking. Histopathologic examinations reveal a strong membranous pattern of E-cadherin (epithelial marker) and absence of vimentin (mesenchymal marker) expression in the normal squamous epithelium of buccal mucosa, contrasting with the loss of E-cadherin expression in human OSCC tissue, which is associated with a poor prognosis [55,56,57]. Downregulation of E-cadherin in OSCC cells leads to poor differentiation, acquisition of invasive properties, and activation of EMT functioning [56]. Thus, it is essential to determine, which factors influence E-cadherin expression in OSCC. p120ctn, a member of the catenin family, binds to E-cadherin to regulate its adhesiveness [58]. However, the functional role of p120ctn extends well beyond this partnering, as the vast majority of OSCCs exhibit loss of membranous p120ctn immunoreactivity and OSCCs with reduced or absent p120ctn expression have a poor prognosis [59]. Indeed, lower levels of E-cadherin and p120 expression are associated with greater OSCC aggressiveness [59]. Our findings are the first to reveal that OSCC-derived WISP-1 promotes activation of Snail via the FAK/ILK/Akt signaling transduction pathways, which leads to Snail nuclear translocation and inhibition of E-cadherin gene transcription. It appears that OSCC-derived WISP-1 is a key regulator of EMT functioning via E-cadherin downregulation.

It is established that miRNAs are important regulators that contribute to EMT functioning through their target genes in cancer cells [21,22]. For instance, miR-300 inhibits the EMT process by increasing E-cadherin expression and decreasing the levels of N-cadherin, vimentin, and Snail in OSCC cells [25]. In lung cancer, periostin promotes Twist and Snail expression by inhibiting miR-381 via the ERK and p38 signaling pathways [60]. Here, we found that miR-153-3p binds directly to the 3’-UTR of Snail and inhibits Snail mRNA translation. OSCC-derived WISP-1 inhibited miR-153-3p expression via the integrin αvβ3 receptor, while miR-153-3p mimic prevented WISP-1-induced enhancement of Snail expression. We did not investigate which upstream mediators influence miR-153-3p expression following WISP-1/integrin αvβ3-induced regulation in OSCC. According to previous research, hsa-miR-153 is an intronic miRNA, with its gene location embedded in *IA-2* (also known as *PTPRN*) and *IA-2β* (also known as *PTPRN2*), with consensus X-Box Binding Protein 1 (XBP1)-binding sites in the promoter regions of *IA-2* and *IA-2β* [61,62]. In the ChIP assay, XBP1 directly binds to the promoter of its host gene *IA-2* to induce miR-153 expression in breast cancer cells under hypoxia [63]. Based on these findings, XBP1 may be a potential upstream mediator of miR-153-3p involved in WISP-1-regulated EMT functioning in OSCC cells.

## 5. Conclusions

**Figure 8:**
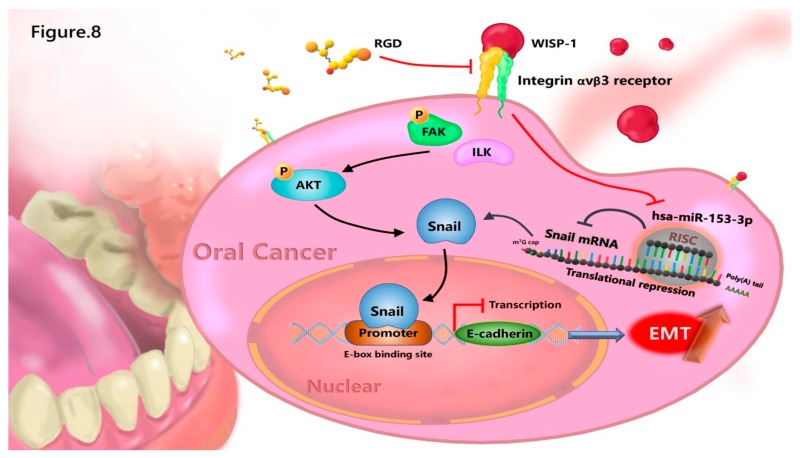
A schematic model depicting how WISP-1 regulates EMT functioning in OSCC cells. The modeling shows two potential signaling pathways underlying OSCC-derived WISP-1 regulation of EMT activation: (1) WISP-1 induces Snail expression via the integrin αvβ3/FAK/ILK/Akt signaling pathway, which in turn activates EMT functioning; (2) WISP-1 also inhibits miR-153-3p-induced downregulation of Snail mRNA translation and thus stimulates Snail protein expression and EMT activation. (RGD peptide: a target epitope of integrin αvβ3)

In summary, we discovered a novel function of WISP-1 in promoting EMT function of OSCC via a specific regulatory mechanism of miR-153-3p/Snail axis. MiR-153-3p directly targets the 3’-UTR of Snail and thereby reduces Snail mRNA translation. OSCC-derived WISP-1 inhibited miR-153-3p through the integrin αvβ3 receptor and subsequently inhibited miR-153-3p-induced inhibition of Snail mRNA translation (Figure 8). High levels of WISP-1 mRNA expression in patients with OSCC were significantly associated with clinical disease stage and regional lymph node metastasis. According to our findings, the inhibition of WISP-1 or overexpression of miR-153-3p may be relevant therapeutic targets in OSCC.

## Supplementary Materials

The following are available online at https://www.mdpi.com/2072-6694/11/12/1903/s1: Figure S1. Analysis of WISP-1 mRNA expression and its regulation of E-cadherin expression. Figure S2. Analysis of TCGA tumor data showing correlations between WISP-1, E-cadherin and Snail mRNA expression. Figure S3. Original unedited blots from primary figures. Figure S4. Original unedited blots from primary figures.

## References

[1] Bagan J., Sarrion G., Jimenez Y. (2010). Oral cancer: Clinical features. Oral Oncol..

[2] Shenoi R., Devrukhkar V., Chaudhuri, Sharma B.K., Sapre S.B., Chikhale A. (2012). Demographic and clinical profile of oral squamous cell carcinoma patients: A retrospective study. Indian J. Cancer.

[3] Asio J., Kamulegeya A., Banura C. (2018). Survival and associated factors among patients with oral squamous cell carcinoma (OSCC) in Mulago hospital, Kampala, Uganda. Cancers Head Neck.

[4] Pires F.R., Ramos A.B., Oliveira J.B., Tavares A.S., Luz P.S., Santos T.C. (2013). Oral squamous cell carcinoma: Clinicopathological features from 346 cases from a single oral pathology service during an 8-year period. J. Appl. Oral Sci..

[5] Rivera C., Venegas B. (2014). Histological and molecular aspects of oral squamous cell carcinoma. Oncol. Lett..

[6] Zini A., Czerninski R., Sgan-Cohen H.D. (2010). Oral cancer over four decades: Epidemiology, trends, histology, and survival by anatomical sites. J. Oral Pathol. Med..

[7] Roche J. (2018). The Epithelial-to-Mesenchymal Transition in Cancer. Cancers.

[8] Kalluri R., Weinberg R.A. (2009). The basics of epithelial-mesenchymal transition. J. Clin. Invest..

[9] Tam W.L., Weinberg R.A. (2013). The epigenetics of epithelial-mesenchymal plasticity in cancer. Nat. Med..

[10] Nieto M.A. (2013). Epithelial plasticity: A common theme in embryonic and cancer cells. Science.

[11] Gonzalez D.M., Medici D. (2014). Signaling mechanisms of the epithelial-mesenchymal transition. Sci. Signal.

[12] Grotegut S., von Schweinitz D., Christofori G., Lehembre F. (2006). Hepatocyte growth factor induces cell scattering through MAPK/Egr-1-mediated upregulation of Snail. EMBO J..

[13] Thiery J.P., Sleeman J.P. (2006). Complex networks orchestrate epithelial-mesenchymal transitions. Nat. Rev. Mol. Cell Biol..

[14] Zhang L., Huang G., Li X., Zhang Y., Jiang Y., Shen J., Liu J., Wang Q., Zhu J., Feng X. (2013). Hypoxia induces epithelial-mesenchymal transition via activation of SNAI1 by hypoxia-inducible factor-1alpha in hepatocellular carcinoma. BMC Cancer.

[15] Diaz V.M., Vinas-Castells R., Garcia de Herreros A. (2014). Regulation of the protein stability of EMT transcription factors. Cell Adh. Migr..

[16] Batlle E., Sancho E., Franci C., Dominguez D., Monfar M., Baulida J., Garcia De Herreros A. (2000). The transcription factor snail is a repressor of E-cadherin gene expression in epithelial tumour cells. Nat. Cell Biol..

[17] Cano A., Perez-Moreno M.A., Rodrigo I., Locascio A., Blanco M.J., del Barrio M.G., Portillo F., Nieto M.A. (2000). The transcription factor snail controls epithelial-mesenchymal transitions by repressing E-cadherin expression. Nat. Cell Biol..

[18] Puisieux A., Brabletz T., Caramel J. (2014). Oncogenic roles of EMT-inducing transcription factors. Nat. Cell Biol..

[19] Nieto M.A., Huang R.Y., Jackson R.A., Thiery J.P. (2016). Emt. 2016. Cell.

[20] Brabletz T., Kalluri R., Nieto M.A., Weinberg R.A. (2018). EMT in cancer. Nat. Rev. Cancer.

[21] Mlcochova H., Machackova T., Rabien A., Radova L., Fabian P., Iliev R., Slaba K., Poprach A., Kilic E., Stanik M. (2016). Epithelial-mesenchymal transition-associated microRNA/mRNA signature is linked to metastasis and prognosis in clear-cell renal cell carcinoma. Sci. Rep..

[22] Legras A., Pecuchet N., Imbeaud S., Pallier K., Didelot A., Roussel H., Gibault L., Fabre E., Le Pimpec-Barthes F., Laurent-Puig P. (2017). Epithelial-to-Mesenchymal Transition and MicroRNAs in Lung Cancer. Cancers.

[23] Zhang J., Ma L. (2012). MicroRNA Control of epithelial-mesenchymal transition and metastasis. Cancer Metastasis Rev..

[24] Jakob M., Mattes L.M., Kuffer S., Unger K., Hess J., Bertlich M., Haubner F., Ihler F., Canis M., Weiss B.G. (2019). MicroRNA expression patterns in oral squamous cell carcinoma: hsa-mir-99b-3p and hsa-mir-100-5p as novel prognostic markers for oral cancer. Head Neck.

[25] Kang Y., Zhang Y., Sun Y., Wen Y., Sun F. (2018). MicroRNA-300 suppresses metastasis of oral squamous cell carcinoma by inhibiting epithelial-to-mesenchymal transition. Onco. Targets Ther..

[26] Wei D., Wang W., Shen B., Zhou Y., Yang X., Lu G., Yang J., Shao Y. (2019). MicroRNA199a5p suppresses migration and invasion in oral squamous cell carcinoma through inhibiting the EMTrelated transcription factor SOX4. Int. J. Mol. Med..

[27] Yap T., Koo K., Cheng L., Vella L.J., Hill A.F., Reynolds E., Nastri A., Cirillo N., Seers C., McCullough M. (2018). Predicting the Presence of Oral Squamous Cell Carcinoma Using Commonly Dysregulated MicroRNA in Oral Swirls. Cancer Prev. Res..

[28] Liu C.J., Kao S.Y., Tu H.F., Tsai M.M., Chang K.W., Lin S.C. (2010). Increase of microRNA miR-31 level in plasma could be a potential marker of oral cancer. Oral Dis..

[29] Yi B., Williams P.J., Niewolna M., Wang Y., Yoneda T. (2002). Tumor-derived platelet-derived growth factor-BB plays a critical role in osteosclerotic bone metastasis in an animal model of human breast cancer. Cancer Res..

[30] Li Z., Zhang L.J., Zhang H.R., Tian G.F., Tian J., Mao X.L., Jia Z.H., Meng Z.Y., Zhao L.Q., Yin Z.N. (2014). Tumor-derived transforming growth factor-beta is critical for tumor progression and evasion from immune surveillance. Asian Pac. J. Cancer Prev..

[31] Lin C.C., Chen P.C., Lein M.Y., Tsao C.W., Huang C.C., Wang S.W., Tang C.H., Tung K.C. (2016). WISP-1 promotes VEGF-C-dependent lymphangiogenesis by inhibiting miR-300 in human oral squamous cell carcinoma cells. Oncotarget.

[32] Chuang J.Y., Chang A.C., Chiang I.P., Tsai M.H., Tang C.H. (2013). Apoptosis signal-regulating kinase 1 is involved in WISP-1-promoted cell motility in human oral squamous cell carcinoma cells. PLoS ONE.

[33] Xu L., Corcoran R.B., Welsh J.W., Pennica D., Levine A.J. (2000). WISP-1 is a Wnt-1- and beta-catenin-responsive oncogene. Genes Dev..

[34] Yang J.Y., Yang M.W., Huo Y.M., Liu W., Liu D.J., Li J., Zhang J.F., Hua R., Sun Y.W. (2015). High expression of WISP-1 correlates with poor prognosis in pancreatic ductal adenocarcinoma. Am. J. Transl. Res..

[35] Nagai Y., Watanabe M., Ishikawa S., Karashima R., Kurashige J., Iwagami S., Iwatsuki M., Baba Y., Imamura Y., Hayashi N. (2011). Clinical significance of Wnt-induced secreted protein-1 (WISP-1/CCN4) in esophageal squamous cell carcinoma. Anticancer Res..

[36] Tsai H.C., Tzeng H.E., Huang C.Y., Huang Y.L., Tsai C.H., Wang S.W., Wang P.C., Chang A.C., Fong Y.C., Tang C.H. (2017). WISP-1 positively regulates angiogenesis by controlling VEGF-A expression in human osteosarcoma. Cell Death Dis..

[37] Lee H.P., Chen P.C., Wang S.W., Fong Y.C., Tsai C.H., Tsai F.J., Chung J.G., Huang C.Y., Yang J.S., Hsu Y.M. (2019). Plumbagin suppresses endothelial progenitor cell-related angiogenesis in vitro and in vivo. J. Funct. Foods.

[38] Liu S.C., Tsai C.H., Wu T.Y., Tsai C.H., Tsai F.J., Chung J.G., Huang C.Y., Yang J.S., Hsu Y.M., Yin M.C. (2019). Soya-cerebroside reduces IL-1 beta-induced MMP-1 production in chondrocytes and inhibits cartilage degradation: Implications for the treatment of osteoarthritis. Food Agr. Immunol..

[39] Lee H.-P., Wang S.-W., Wu Y.-C., Tsai C.-H., Tsai F.-J., Chung J.-G., Huang C.-Y., Yang J.-S., Hsu Y.-M., Yin M.-C. (2019). Glucocerebroside reduces endothelial progenitor cell-induced angiogenesis. Food Agric. Immunol..

[40] Yang W.H., Chang A.C., Wang S.W., Wang S.J., Chang Y.S., Chang T.M., Hsu S.K., Fong Y.C., Tang C.H. (2016). Leptin promotes VEGF-C production and induces lymphangiogenesis by suppressing miR-27b in human chondrosarcoma cells. Sci. Rep..

[41] Brigstock D.R. (2003). The CCN family: A new stimulus package. J. Endocrinol..

[42] Holbourn K.P., Perbal B., Ravi Acharya K. (2009). Proteins on the catwalk: Modelling the structural domains of the CCN family of proteins. J. Cell Commun. Signal..

[43] Wu C.L., Tsai H.C., Chen Z.W., Wu C.M., Li T.M., Fong Y.C., Tang C.H. (2013). Ras activation mediates WISP-1-induced increases in cell motility and matrix metalloproteinase expression in human osteosarcoma. Cell. Signal.

[44] Takada Y., Ye X., Simon S. (2007). The integrins. Genome Biol..

[45] Hehlgans S., Haase M., Cordes N. (2007). Signalling via integrins: Implications for cell survival and anticancer strategies. Biochim. Biophys. Acta.

[46] Shishido S., Bonig H., Kim Y.M. (2014). Role of integrin alpha4 in drug resistance of leukemia. Front. Oncol..

[47] Serrano-Gomez S.J., Maziveyi M., Alahari S.K. (2016). Regulation of epithelial-mesenchymal transition through epigenetic and post-translational modifications. Mol. Cancer.

[48] Bettendorf O., Piffko J., Bankfalvi A. (2004). Prognostic and predictive factors in oral squamous cell cancer: Important tools for planning individual therapy?. Oral Oncol..

[49] Parkin D.M., Bray F., Ferlay J., Pisani P. (2005). Global cancer statistics, 2002. CA Cancer J. Clin..

[50] Jung E.K., Kim S.A., Yoon T.M., Lee K.H., Kim H.K., Lee D.H., Lee J.K., Chung I.J., Joo Y.E., Lim S.C. (2017). WNT1-inducible signaling pathway protein-1 contributes to tumor progression and treatment failure in oral squamous cell carcinoma. Oncol. Lett..

[51] Lau H.K., Wu E.R., Chen M.K., Hsieh M.J., Yang S.F., Wang L.Y., Chou Y.E. (2017). Effect of genetic variation in microRNA binding site in WNT1-inducible signaling pathway protein 1 gene on oral squamous cell carcinoma susceptibility. PLoS ONE.

[52] Clausen M.J., Melchers L.J., Mastik M.F., Slagter-Menkema L., Groen H.J., van der Laan B.F., van Criekinge W., de Meyer T., Denil S., Wisman G.B. (2016). Identification and validation of WISP1 as an epigenetic regulator of metastasis in oral squamous cell carcinoma. Genes Chromosomes Cancer.

[53] Lamouille S., Xu J., Derynck R. (2014). Molecular mechanisms of epithelial-mesenchymal transition. Nat. Rev. Mol. Cell Biol..

[54] Gavert N., Ben-Ze’ev A. (2008). Epithelial-mesenchymal transition and the invasive potential of tumors. Trends Mol. Med..

[55] Balasundaram P., Singh M.K., Dinda A.K., Thakar A., Yadav R. (2014). Study of beta-catenin, E-cadherin and vimentin in oral squamous cell carcinoma with and without lymph node metastases. Diagn. Pathol..

[56] Mehendiratta M., Solomon M.C., Boaz K., Guddattu V., Mohindra A. (2014). Clinico-pathological correlation of E-cadherin expression at the invasive tumor front of Indian oral squamous cell carcinomas: An immunohistochemical study. J. Oral Maxillofac. Pathol..

[57] Krisanaprakornkit S., Iamaroon A. (2012). Epithelial-mesenchymal transition in oral squamous cell carcinoma. ISRN Oncol..

[58] Lo Muzio L., Pannone G., Santarelli A., Bambini F., Mascitti M., Rubini C., Testa N.F., Dioguardi M., Leuci S., Bascones A. (2013). Is expression of p120ctn in oral squamous cell carcinomas a prognostic factor?. Oral Surg. Oral Med. Oral Pathol. Oral Radiol..

[59] Jiang Y., Liao L., Shrestha C., Ji S., Chen Y., Peng J., Wang L., Liao E., Xie Z. (2015). Reduced expression of E-cadherin and p120-catenin and elevated expression of PLC-gamma1 and PIKE are associated with aggressiveness of oral squamous cell carcinoma. Int. J. Clin. Exp. Pathol..

[60] Hu W.W., Chen P.C., Chen J.M., Wu Y.M., Liu P.Y., Lu C.H., Lin Y.F., Tang C.H., Chao C.C. (2017). Periostin promotes epithelial-mesenchymal transition via the MAPK/miR-381 axis in lung cancer. Oncotarget.

[61] Xu H., Abuhatzira L., Carmona G.N., Vadrevu S., Satin L.S., Notkins A.L. (2015). The Ia-2beta intronic miRNA, miR-153, is a negative regulator of insulin and dopamine secretion through its effect on the Cacna1c gene in mice. Diabetologia.

[62] Mandemakers W., Abuhatzira L., Xu H., Caromile L.A., Hebert S.S., Snellinx A., Morais V.A., Matta S., Cai T., Notkins A.L. (2013). Co-regulation of intragenic microRNA miR-153 and its host gene Ia-2 beta: Identification of miR-153 target genes with functions related to IA-2beta in pancreas and brain. Diabetologia.

[63] Liang H., Xiao J., Zhou Z., Wu J., Ge F., Li Z., Zhang H., Sun J., Li F., Liu R. (2018). Hypoxia induces miR-153 through the IRE1alpha-XBP1 pathway to fine tune the HIF1alpha/VEGFA axis in breast cancer angiogenesis. Oncogene.

