# Supplementary material for: WISP-1 Promotes Epithelial-Mesenchymal Transition in Oral Squamous Cell Carcinoma Cells via the miR-153-3p/Snail Axis
Source: Cancers (Basel). 2019 Nov 29;11(12):1903. doi: 10.3390/cancers11121903 (PMC6966565; doi:10.3390/cancers11121903)
Supplement: Supplementary file 1 [file cancers-11-01903-s001.pdf]

**Supplementary data**

**(A)**

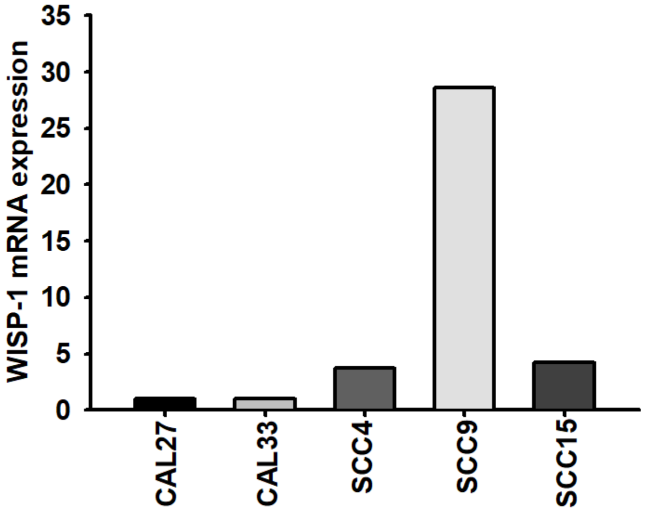


**(B)**

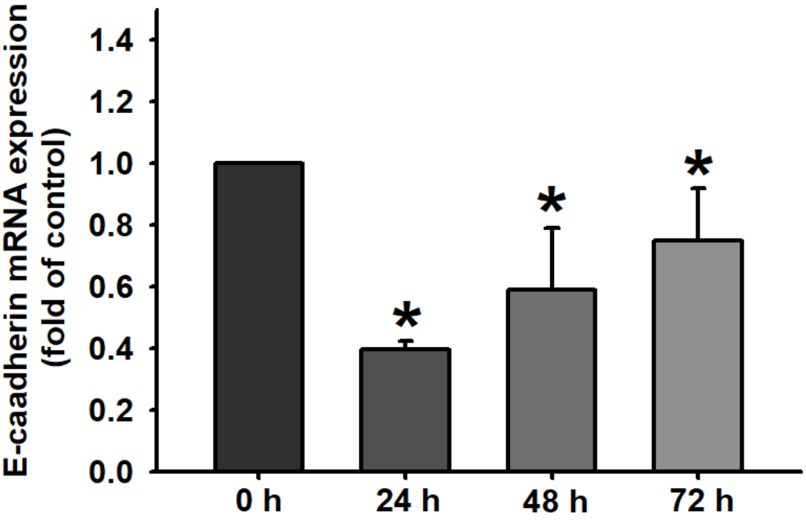


**Figure S1. Analysis of WISP-1 mRNA expression and its regulation of E-cadherin expression.** (A) Levels of WISP-1 mRNA expression in different OSCC cell lines from the CCLE database. (B) Levels of E-cadherin mRNA expression after 0, 24, 48, or 72 h of WISP-1 treatment. Results are expressed as the mean ± SEM. *$p < 0.05$ compared with the 0 h group.

**(A)**

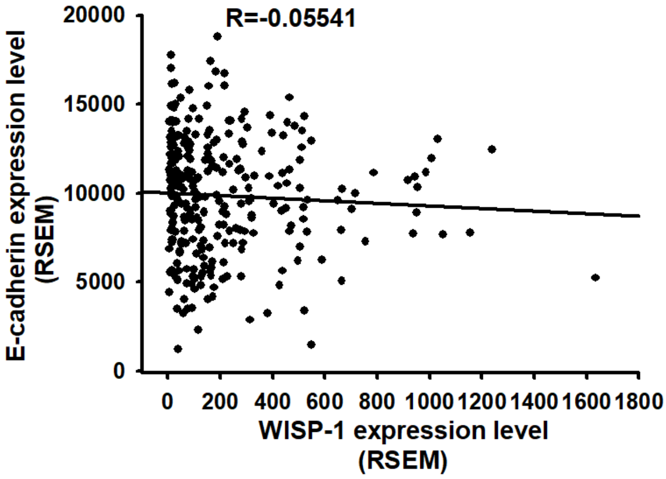


**(B)**

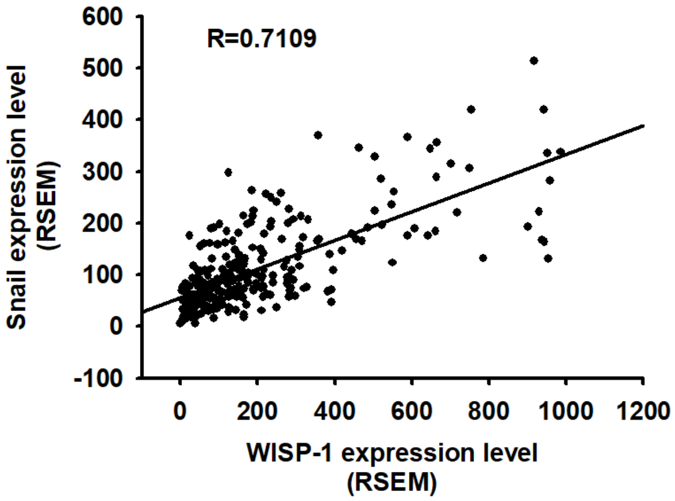


**Figure S2. Analysis of TCGA tumor data showing correlations between WISP-1, E-cadherin and Snail mRNA expression.** (A) Correlation analysis of WISP-1 and E-cadherin mRNA expression. (B) Correlation analysis of WISP-1 and Snail mRNA expression.

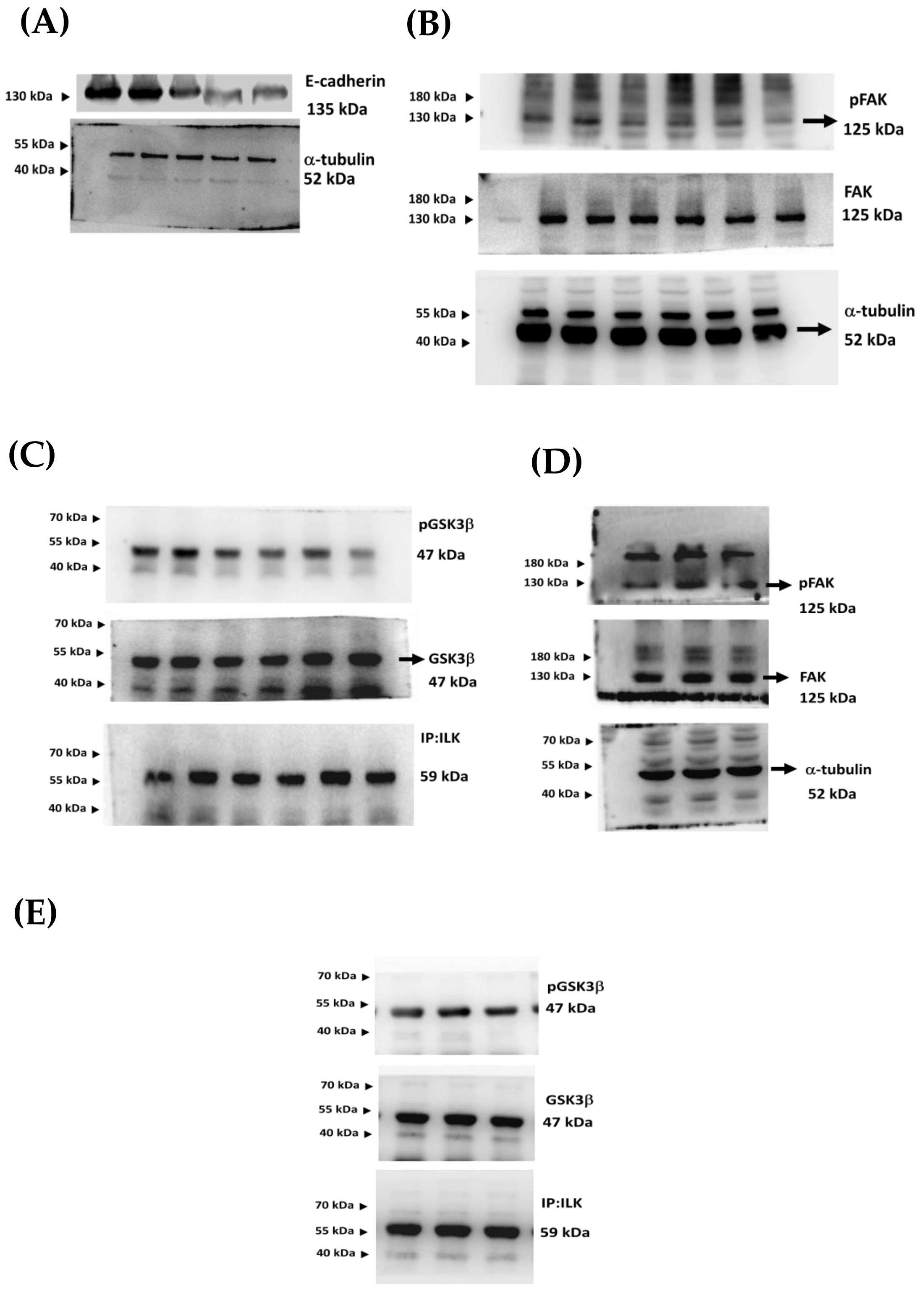


**Figure S3. Original unedited blots from primary figures.** (A) Figure 2D, (B) Figure 4A, (C) Figure 4B, (D) Figure 4C, (E) Figure 4D.

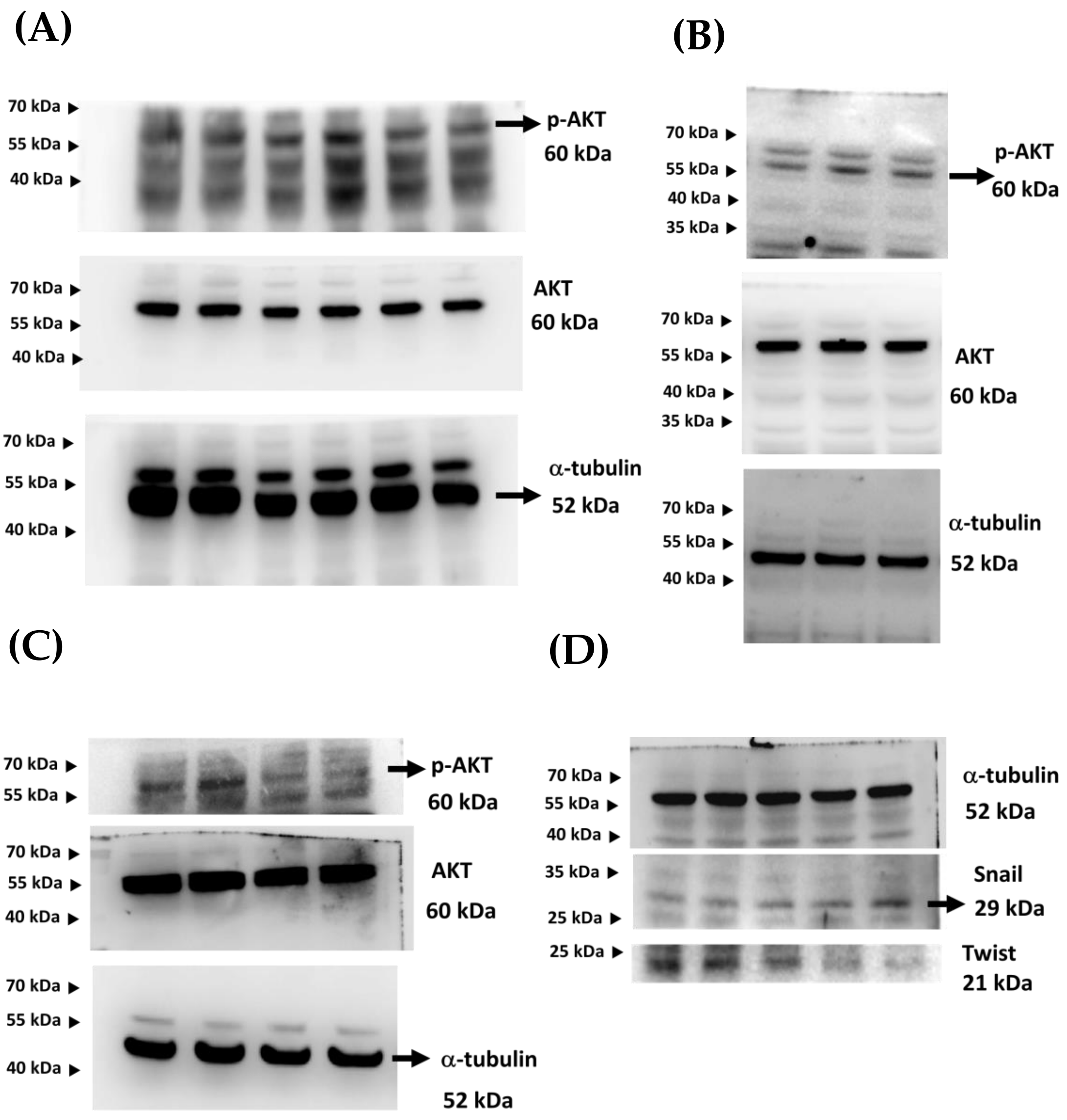


**Figure S4. Original unedited blots from primary figures.** (A) Figure 5A, (B) Figure 5B, (C) Figure 5C, (D) Figure 6D.
